# Genetic alterations in main candidate genes during melanoma progression

**DOI:** 10.18632/oncotarget.23989

**Published:** 2018-01-03

**Authors:** Maria Cristina Sini, Valentina Doneddu, Panagiotis Paliogiannis, Milena Casula, Maria Colombino, Antonella Manca, Gerardo Botti, Paolo A. Ascierto, Amelia Lissia, Antonio Cossu, Giuseppe Palmieri

**Affiliations:** ^1^ Unit of Cancer Genetics, Institute of Biomolecular Chemistry, National Research Council, Sassari, Italy; ^2^ Department of Surgical, Microsurgical and Medical Sciences, University of Sassari, Sassari, Italy; ^3^ Department of Clinical and Experimental Medicine, University of Sassari, Sassari, Italy; ^4^ Istituto Nazionale Tumori, Fondazione Pascale, Napoli, Italy

**Keywords:** malignant melanoma, fluorescence in situ hybridization (FISH) analysis, genetic heterogeneity, mutation analysis, oncogenic driver genes

## Abstract

Cutaneous melanoma is a common and aggressive human skin cancers. Much is actually known about the molecular mechanisms underlying melanoma pathogenesis. The aim of the study was to evaluate any possible correlation between mutations in main growth-controlling genes (*BRAF, NRAS, CDKN2A*) and copy number variations in frequently amplified candidate genes (*MITF, EGFR, CCND1, cMET,* and *cKIT*) during melanoma initiation and progression.

A large series of primary and secondary melanoma tissue samples (*N* = 274) from 232 consecutively-collected patients of Italian origin as well as 32 tumor cell lines derived from primary and metastatic melanomas underwent mutation screening and fluorescence in situ hybridization (FISH) analysis. Overall, *BRAF, NRAS,* and *CDKN2A* were found mutated in 62.5%, 12.5% and 59% cell lines and in 47%, 16%, 12% tumor tissues, respectively. Quite identical mutation patterns between primary tumors and metastatic lesions were found for *BRAF* and *NRAS* genes; mutations of *CDKN2A* gene appeared to be instead selected during tumor progression. In cell lines, high rates of gene amplifications were observed (varying from 12.5% for *cKIT* to 50% for *MITF*); vast majority of cell lines (75%) presented at least one amplified gene. Conversely, prevalence of gene amplification was significantly and progressively decreasing in melanoma metastases (12%) and primary melanomas (4%). Our findings suggest that gene amplifications may be acquired during the late phases of melanoma evolution and mostly act as “passenger” or “non-causative” alterations.

## INTRODUCTION

Malignant cutaneous melanoma (MCM) is one of the most frequent and aggressive forms of skin cancer which arises from epidermal melanocytes and occurs in all age groups [1]. Several risk factors for development of MCM have been identified; a light skin phototype, an excessive sun exposure and/or increased incidence of sunburns, a large number of acquired common nevi, the occurrence of atypical nevi and a family history of melanoma have been associated with a higher risk for its disease [2, 3]. The MCM incidence has significantly increased in recent years, especially in white populations, despite the efforts to prevent excessive sun exposure; in 2012, more than 230,000 new cases and 55,000 deaths were estimated worldwide [4, 5]. Although at a lesser extent, mortality rates have also risen in the last decade worldwide; in the USA, the raw mortality rates per 100,000 inhabitants per year increased from 2.8 to 3.1, with an estimate of 10,130 deaths from melanoma in 2016 (they were 8650 in 2009) [6]. These figures evidence the need to enhance prevention and surveillance strategies as well as to improve the available treatments, especially for the cases with advanced disease characterized by poor survival rates [7].

Knowledge of the molecular mechanisms involved in melanoma development and progression is crucial for both classification and management of the disease. It is well known that mutations in main candidate genes concur to the carcinogenesis and the dissemination of the disease. *BRAF* and *NRAS* genes, which encode proteins belonging to the mitogen-activated protein kinase (MAPK) signal transduction pathway, have been found to play an essential role in large part of MCMs. In particular, the *BRAF* gene has been found mutated in 40-60% of cases, with the most prevalent mutation (about 90% of cases) being the replacement of glutamic acid with valine at codon 600 (BRAF^V600E^) [8]. This variant, as other mutations in the *BRAF* kinase domain, leads to a continuous stimulation of cell proliferation and tumor growth through constitutive phosphorylation of ERK. Nevertheless, the identification of *BRAF* mutations in common nevi suggests that its activation is not sufficient by itself for the development of MCM [9]. On the other hand, *NRAS* is the gene most involved in MCM among the three members of the RAS family, and acts through activation of specific cytoplasmic proteins downstream: RAF and phosphatidylinositol 3 kinase (PI3K) [9, 10]. Also the CDKN2A-dependent pathway has been found to be involved in the genesis of MCM. *CDKN2A* is a tumor suppressor gene and inactivating mutations in this gene are 7-10 times more frequent in patients with a strong family history of melanoma, in comparison to those with sporadic melanoma. Genetic alterations of *CDKN2A* cause loss of inhibition of the protein kinase cyclin-dependent kinase 4 (CDK4)/Cyclin D1 (CCND1), which affects the cell-cycle progression depending on the retinoblastoma susceptibility (RB) protein [9, 11]. Furthermore, the occurrence of *BRAF* mutations enhances the expression of *CDKN2A* activating a defensive mechanism with induction of cellular senescence and cell-cycle arrest [12]. These observations constitute the basis of the current molecular classification of MCM.

Less clear is the role of the aforementioned genetic alterations in the progression of the disease, though *BRAF* and *NRAS* mutations have been demonstrated to present high rates of consistency between primary and metastatic melanomas [13, 14]. Amplification of *CCND1*, which is usually observed in primary MCM on skin chronically exposed to sun presenting low rates of *BRAF* mutations, has been described associated with *BRAF* mutations in metastatic lesions [15]. Analogously, *cKIT* gene was found amplified in 2–7% of primary MCM, with higher amplification in metastatic than in primary lesions [13]. Moreover, the amplification of *microphthalmia-associated transcription factor* (*MITF*) gene seems to play a role in melanoma progression [16, 17], while the role of the genomic amplification for *EGFR* and *cMET* genes is less clear [18]. Finally, little is known on the interactions between mutations and amplifications of such candidate genes into the initiation and progression of the MCM lesions.

For this reason, we planned molecular investigations in tumor cell lines and human neoplastic tissues obtained by primary and metastatic MCMs, originating from the same patients in a subset of cases. In particular, we evaluated the spectrum and distribution of *BRAF*, *NRAS* and *CDKN2A* gene mutations as well as the amplification levels of *MITF*, *EGFR*, *CCND1*, *cKIT*, and *cMET* genes in order to further assess their involvement during the genesis and dissemination of MCM.

## RESULTS

### *BRAF*, *NRAS* and *CDKN2A* mutations in cell lines

A series of 32 melanoma cell lines - eight derived from surgically-excised primary melanomas, seventeen from cutaneous or lymph nodal metastases - were firstly investigated for mutations in the three main genes (*BRAF*, *NRAS*, and *CDKN2A*) involved in melanoma pathogenesis (Table 1). *BRAF* was found mutated in 20/32 (62.5%) cell lines, with identical distribution of mutation frequencies between those derived from primary (5/8; 62.5%) and those derived from metastatic (15/24; 62.5%) melanomas. According with the literature [9], the most frequent *BRAF* mutation (14/20; 70.0%) was the substitution of valine by a glutamic acid at position 600 (V600E). A much lower (4/32; 12.5%) prevalence of *NRAS* mutations was instead observed; again, no difference in mutation frequency distribution among cells lines from primary (1/8; 12.5%) versus metastatic (3/24; 12.5%) melanomas was registered. No concomitant mutation in *BRAF* and *NRAS* genes was detected (Table 1). Conversely, the prevalence of *CDKN2A* alterations (19/32; 59.4%)—which include inactivating gene mutations, exon deletions, and amino acid substitutions—was much higher in cell lines derived from melanoma metastases (16/24; 66.7%) than in those derived from primary melanomas (3/8; 37.5%) (Table 1).

**Table 1:** Mutations detected in candidate genes (*BRAF, NRAS*, and *CDKN2A*) among melanoma cell lines derived from primary and metastatic melanomas

Cell line origin	BRAF	NRAS	CDKN2A
***Primary melanoma***			
**GR-Mel**			
**LCP-Mel**	V600R		del ex 2
**MNG-Mel**			
**PNP-Mel**	V600E		G101W
**SBCL2**		Q61L	del ex 1, 2, 3
**ST-Mel**	G466E		
**UACC 62**	V600E		
**WM-115-4**	V600D		
***Cutaneous melanoma metastasis***			
**397-Mel**			del ex 2
**LB-24-Mel**			
**M14**	V600E		455insCdel26 IVS1+2T>C
**PR-Mel**	V600R		
**SN-Mel**	V600E		
**WM-266-4**	V600D		del ex 1
**PE-MEL-41**	V600E		W110^*^ A148T
**PE-MEL-43**	V600E		W110^*^ A148T
**PE-MEL-47**	V600E		W110^*^ A148T
***Lymph node melanoma metastasis***			
**13443-Mel**			
**CN-Mel**		Q61R	
**CR-Mel**		Q61K	del ex 2
**GL-Mel**			del ex 2
**LCM-Mel**	V600R		del ex 1, 2
**MAR-Mel**			
**SK-Mel-28**	V600E		
**COPA 159**	V600E		del ex 1,2,3
**A375**	V600E		E61^*^; E69^*^
**PNM-Mel**	V600E		G101W
**Mel 3.0**		Q61L	del ex 1, 2, 3
**INT 9009**	V600E		del ex 1, 2, 3
**MALME 37**	V600E		del ex 1, 2, 3
**ME 33797**	V600E		
**UACC 257**			del ex 1, 2, 3

Abbreviations: del, deletion; ex, exon.

### Gene amplifications in melanoma cell lines

Melanoma cell lines were investigated for the ploidy pattern at genomic loci of five candidate genes involved in melanocytic transformation and progression by a two-colour fluorescence *in situ* hybridization (FISH) analysis, using five genomic subclones as probes spanning the following chromosomal regions: *MITF* at chromosome 3p14.1, *EGFR* at 7p11.2, *CCND1* at 11q13.2, *cMET* at 7q31.2, and *cKIT* at 4q12.

The *MITF* gene presented the highest frequency of genomic amplification, with 16/32 (50%) altered cell lines (Table 2; Supplementary Table 1). Amplification of *MITF* gene was observed in the primary melanoma cell lines (2/8; 25%) at significantly lower level than that observed in metastatic melanoma cell lines (14/24; 58.3%) (*p* = 0.023; Chi-square).

**Table 2:** Amplification (AMPL) in candidate genes (*MITF, EGFR, CCND1, cMET,* and *cKIT*) among melanoma cell lines derived from primary and metastatic melanomas

Cell line	Tissue origin	MITF	EGFR	CCND1	cMET	cKIT
*GR-Mel*	primary melanoma	Disomy	Disomy	**AMPL**	Disomy	Disomy
*LCP-Mel*	primary melanoma	Disomy	Disomy	Disomy	Disomy	Disomy
*MNG-Mel*	primary melanoma	Disomy	Disomy	Disomy	Disomy	Disomy
*PNP-Mel*	primary melanoma	Disomy	Disomy	Disomy	Disomy	Disomy
*WM-115-4*	primary melanoma	Disomy	**AMPL**	**AMPL**	Disomy	Disomy
*ST-Mel*	primary melanoma	**AMPL**	Disomy	Disomy	Disomy	Disomy
*UACC 62*	primary melanoma	Disomy	Disomy	Disomy	Disomy	Disomy
*SBCL2*	primary melanoma	**AMPL**	**AMPL**	Disomy	Disomy	Disomy
*COPA 159*	melanoma metastasis	**AMPL**	Disomy	Disomy	Disomy	Disomy
*PE-MEL-41*	melanoma metastasis	**AMPL**	Disomy	Disomy	**AMPL**	Disomy
*PE-MEL-43*	melanoma metastasis	**AMPL**	Disomy	Disomy	Disomy	Disomy
*PE-MEL-47*	melanoma metastasis	**AMPL**	Disomy	AMPL	Disomy	Disomy
*PNM-Mel*	melanoma metastasis	Disomy	**AMPL**	Disomy	**AMPL**	Disomy
*WM-266-4*	melanoma metastasis	**AMPL**	Disomy	Disomy	Disomy	**AMPL**
*UACC 257*	melanoma metastasis	**AMPL**	**AMPL**	**AMPL**	Disomy	**AMPL**
*ME 33797*	melanoma metastasis	**AMPL**	Disomy	Disomy	Disomy	Disomy
*A375*	melanoma metastasis	Disomy	Disomy	Disomy	Disomy	Disomy
*Mel3.0*	melanoma metastasis	**AMPL**	Disomy	Disomy	Disomy	Disomy
*LB-24-Mel*	melanoma metastasis	**AMPL**	**AMPL**	**AMPL**	**AMPL**	Disomy
*PR-Mel*	melanoma metastasis	**AMPL**	Disomy	Disomy	Disomy	Disomy
*SN-Mel*	melanoma metastasis	Disomy	**AMPL**	Disomy	Disomy	Disomy
*MALME 37*	melanoma metastasis	Disomy	**AMPL**	Disomy	Disomy	Disomy
*INT 9009*	melanoma metastasis	Disomy	**AMPL**	Disomy	Disomy	Disomy
*M14*	melanoma metastasis	Disomy	Disomy	Disomy	Disomy	Disomy
*397-Mel*	melanoma metastasis	**AMPL**	Disomy	Disomy	Disomy	Disomy
*GL-Mel*	melanoma metastasis	Disomy	Disomy	Disomy	Disomy	Disomy
*CN-Mel*	melanoma metastasis	Disomy	Disomy	Disomy	Disomy	Disomy
*CR-Mel*	melanoma metastasis	**AMPL**	Dysomy	**AMPL**	Disomy	Disomy
*13443-Mel*	melanoma metastasis	Disomy	**AMPL**	Disomy	**AMPL**	**AMPL**
*MAR-Mel*	melanoma metastasis	**AMPL**	**AMPL**	**AMPL**	**AMPL**	Disomy
*SK-Mel-28*	melanoma metastasis	**AMPL**	**AMPL**	**AMPL**	**AMPL**	**AMPL**
*LCM-Mel*	melanoma metastasis	Disomy	**AMPL**	Disomy	**AMPL**	Disomy

Interphase melanoma cell lines showed a similar high level of amplification for *EGFR* gene (12/32; 37.5%) (Table 2; Supplementary Table 1); again, cell lines derived from primary melanoma (2/8; 25%) presented a significantly lower level of gene amplification compared to those derived from metastatic melanoma (10/24; 41.7%) (*p* = 0.041; Chi-square).

Amplification frequency was instead found at intermediate levels for *CCND1* (8/32; 25%) and *cMET* (7/32; 21.9%) genes, with the lowest rate of gene amplification observed for cKIT (4/32; 12.5%) (Table 2; Supplementary Table 1). With exclusion of the *CCND1* amplification, which was equally distributed between cell lines from primary and metastatic melanomas [2/8 (25%) vs. 6/24 (25%)], *cMET* and *cKIT* amplifications were detected only in cell lines from melanoma metastases (Table 2; Supplementary Table 1).

Overall, vast majority of cell lines (24/32; 75%) presented at least one amplified gene, with about one tenth of them (4/32; 12.5%) carrying four or five amplified genes (Table 2; Supplementary Table 1).

### Gene mutations and amplifications in melanoma tissues

To evaluate the distribution of gene mutation and amplification *in vivo*, a total of 274 tumor tissues (124 primary melanomas and 150 melanoma metastases) from 232 patients was collected and included into the study.

All somatic samples were firstly screened for *BRAF* and *NRAS* mutations and results are reported in Table 3. Briefly, 58/124 (46.8%) primary melanomas and 70/150 (46.7%) metastatic tissues presented mutations in *BRAF* gene, whereas *NRAS* mutations were found in 19/124 (15.3%) primary tumors and 24/150 (16.0%) melanoma metastases (Table 3). The BRAF^V600E^ variant was the most represented *BRAF* mutation (115/128; 89.8%); all *NRAS* mutations were found at the codon 61 of the gene (Table 3). Again, neither coexistent *BRAF*/*NRAS* mutations nor different mutation frequency distributions between primary and metastatic melanomas were observed. Among available DNA samples, the rate of mutations in *CDKN2A* was much higher in melanoma metastases (11/72; 15.3%) versus primary melanomas (4/54; 7.4%) (data not shown).

**Table 3:** Distribution and types of *BRAF* and *NRAS* mutations in primary and metastatic tumor tissues from cutaneous melanoma patients

Sample	No. of samples	Frequency of mutations and subtypes, n (%)		
		*BRAF* mutation	*NRAS* mutation	*BRAF* or *NRAS* mutation
Primary melanoma	124	58 (47) *53 V600E* *4 V600K* *1 V600D*	19 (15) *12 Q61R* *5 Q61L* *2 Q61K*	77 (62)
Melanoma metastasis	150	70 (47) *62 V600E* *6 V600K* *2 V600D*	24 (16) *16 Q61R* *7 Q61L* *1 Q61K*	94 (63)
Total samples	274	128 (47) *115 V600E* *10 V600R* *3 V600D*	43 (16) *28 Q61L* *12 Q61K* *3 Q61R*	171 (62)

**Figure 1:**
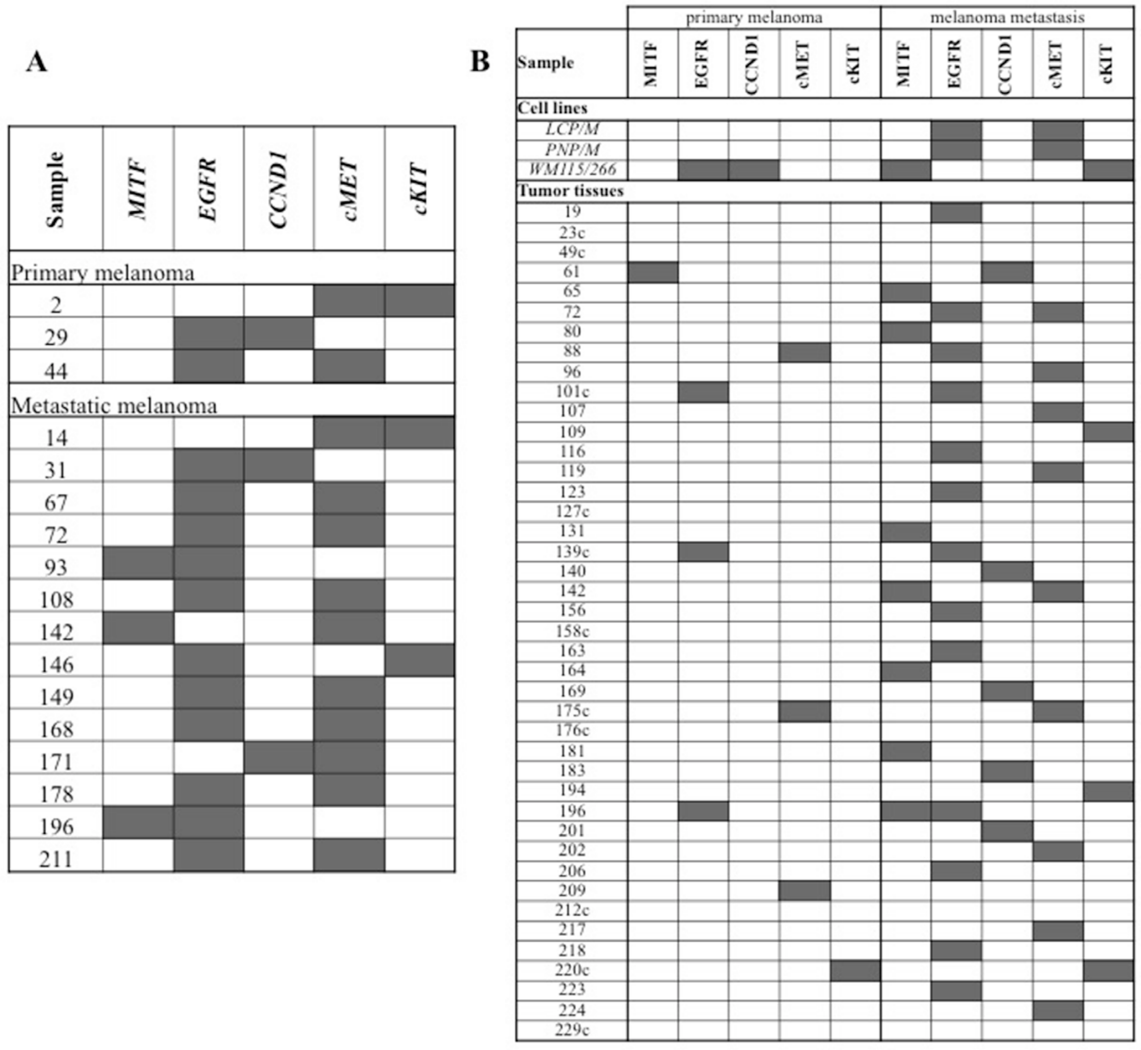
Distribution of gene amplifications in melanoma samples (**A**) Tumor tissues presenting multiple gene amplifications. (**B**) Paired melanoma samples from the same patients. Individual gene amplifications (gray squares) are shown across all cases. The patients with consistent patterns of gene amplification between primary and metastatic melanoma tissues are indicated by the suffix “c”.

Paraffin-embedded nuclei from 262 melanoma tissue sections were assessed for amplification in all five candidate genes (*MITF*, *EGFR*, *CCND1*, *cMET*, and *cKIT*). Indeed, FISH analysis failed to produce a detectable result in limited fractions of tumor tissues for each gene (ranging from 6 tissue samples for *cKIT* to 12 for *CCND1*) (Table 4). Considering the total amount of tissue samples analyzed by FISH (*N* = 1323), 112 (8.5%) melanoma tissues were found to carry amplification at one or more loci spanning our candidate genes (Table 4). In Figure 1A, the very low amount (14/1323; 1.1%) of melanoma tissues presenting with more than one amplified gene signal is reported. Overall, prevalence of gene amplification was significantly lower in primary melanomas (24/592; 4.1%) than in melanoma metastases (88/731; 12.0%) (*p* = 0.007; Table 4).

**Table 4:** Distribution of gene amplifications in primary and metastatic tumor tissues from cutaneous melanoma patients

Sample	MITF	EGFR	CCND1	cMET	cKIT
Primary melanomas	3/118	9/119	1/118	7/117	4/120
	*(2.5 %)*	*(7.6 %)*	*(0.8 %)*	*(6.0 %)*	*(3.3 %)*
Metastatic melanomas	15/146	39/145	14/144	26/148	8/148
	*(10.3 %)*	*(26.9 %)*	*(9.7 %)*	*(17.6 %)*	*(5.4 %)*
All types	18/264	48/264	15/262	33/265	12/268
	*(6.8 %)*	*(18.2 %)*	*(5.7 %)*	*(12.5 %)*	*(4.5 %)*
*p-value (primary vs. metastasis)*	*< 0.01*	*< 0.01*	*< 0.01*	*0.019*	*0.044*

### Gene amplification pattern in primary and metastatic melanoma from the same patient

Among the 232 patients of our series, paired samples of primary melanomas and synchronous or asynchronous metastases were obtained from about one fifth of cases (42/232; 18.1%), whereas primary and metastatic tumor tissues represented the only available specimens in about one third (82/232; 35.3%) and half (108/232; 46.6%) of the cases, respectively. Among the 42 patients with paired primary and secondary melanoma samples (Figure 1B), the rate of gene amplification was significantly higher in melanoma metastases (37/210 total analyzed samples; 17.6%) than in primary melanomas (8/210; 3.8%) (*p* < 0.001). Overall, the increased copy number of candidate genes seems to represent an alteration selected during tumor progression. Including the three melanoma cell lines derived from the same patients, the rate of consistency for gene amplification patterns between primary and secondary tumor samples was 24.4% (11/45; Figure 1B).

### Correlation between gene amplifications and mutations in main candidate genes

No difference was instead detected between occurrence of gene amplification and *BRAF* mutation status both *in vitro* (see Tables 1 and 2) and *in vivo* (data not shown): 15/20 (75%) *BRAF*-mutated and 9/12 (75%) *BRAF*-wild-type cell lines as well as 57/576 (9.9%) *BRAF*-mutated and 59/683 (8.6%) *BRAF*-wild-type melanoma tissues were found to carry amplification in at least one gene locus. The large increase of gene amplification moving from the *in vivo* melanoma tissues to the *in vitro* cultured melanoma cells%—which represent the highest malignant phenotype—is a further indication that copy number of such candidate genes is steadily increased during tumor progression. For melanoma cell lines, the same lack of correlation between gene amplification and *CDKN2A* mutational status was observed (see Tables 1 and 2): 14/19 (74%) *CDKN2A*-mutated and 10/13 (77%) *CDKN2A*-wild-type cell lines were found to carry amplification in at least one gene locus. No correlation was possible to infer into the group with *NRAS* mutations, due to the low number of *NRAS*-mutated cases.

## DISCUSSION

In this study, we tried to assess the existence of any correlation between mutations in the main genes involved in melanoma initiation and progression (*BRAF*, *NRAS*, *CDKN2A*) and copy number variations in a subset of candidate genes (*MITF*, *EGFR*, *CCND1*, *cMET*, and *cKIT*) already demonstrated at single level to be amplified during melanoma pathogenesis. For this purpose, we used a large series of melanoma tissue samples (*N* = 274)—almost equally distributed between primary (45%) and secondary (55%) melanomas—from 232 consecutively-collected patients of the Italian population as well as a representative group of 32 tumor cell lines generated from cultured primary melanomas and melanoma metastases. To our knowledge, this is the first population-based report simultaneously comparing all these alterations in such specific genes highly pathogenic for melanoma.

The prevalence of *BRAF* and *NRAS* mutations in cell lines and tumor tissues from MCM patients was similar with that reported in the scientific literature and other previous reports published by our group [13–14, 19]. Furthermore, *BRAF* and *NRAS* mutations were confirmed to be mutually exclusive, with a good consistency in mutation distribution between primary and metastatic melanoma lesions. However, the demonstration that significant mutation discrepancies occur in multiple melanomas from the same patients [13] outlines the complexity of the pathogenetic mechanisms involved into the development of MCM, which in turn might impact both the grade and duration of the responsiveness to therapy with BRAF and MEK inhibitors [20].

Mutations inactivating *CDKN2A* represent an independent oncogenic mechanism in MCM initiation and progression through the loss of inhibition of the proliferation-controlling CCND1-RB signaling pathway [9, 11]. In our series, occurrence of *CDKN2A* gene alterations in melanoma tissues and cell lines appears to consistently increase moving from primary tumors to metastases till to cultured melanoma cells. This is consistent with the findings demonstrating a progressive inactivation of the *CDKN2A* tumor suppressor gene during melanoma progression, which in turn promotes uncontrolled cell proliferation, tumor growth, and increased aggressiveness of tumor cells [9, 11].

Regarding the amplification of the five genes studied, the vast majority of cell lines (75%) presented at least one amplified gene, with about one tenth of them carrying four or five amplified genes, while in human MCM tissues, less than one tenth (8.5%) of cases was found to carry amplification at one or more loci spanning the candidate genes, all of them observed in metastatic lesions. These figures underline the relevance of the genomic amplification in the late progression and dissemination of the MCM disease.

### Here we briefly went through the different types of gene amplifications (see Table 4)

In recent reports, 10–15% and 15–20% of human MCMs show *MITF* gene amplification in primary and metastatic melanomas, respectively, correlated with a decrease in 5-year patient survival [21, 22]. In our study, much lower levels of gene amplification were detected for the *MITF*, with a statistically significant difference between frequencies in primary and metastatic lesions (2.5% and 10.3%, respectively). MITF is a lineage restricted basic helix-loop-helix leucine zipper transcription factor essential for melanocyte development, differentiation, and survival [22]. *BRAF* mutation and *CDKN2A* inactivation have been found associated with *MITF* amplification in melanoma cell lines [9, 11, 22], but such a correlation was not confirmed in our series. Nevertheless, it has been shown that oncogenic activation of upstream MAPK components is associated with a marked degradation of MITF; therefore, the intracellular levels of MITF should depend on the activation status of the *BRAF* gene [22]. On this regard, low intracellular levels of the MITF protein have been reported in invasive lesions and were correlated with faster progression and a worse prognosis of the disease [23]. For this reason the amplification of the *MITF* gene, with or without alterations of the corresponding protein expression levels, indicate patients who may benefit of treatment with inhibitors of histone deacetylase (HDAC), able to interfere with the expression of MITF protein [24]. To date, no predictive role for response to therapy has been documented, whereas *MITF* amplification has been implied on prediction of disease melanoma progression and disease prognosis [16–17].

The *EGFR* gene was found amplified in 37.5% of cell lines and 18% of MCM tissues, again with rates significantly higher in metastases than in primary lesions. The epidermal growth factor receptor (*EGFR*, *ErbB1*, or *HER1*) gene is located at chromosomal region 7p11.2 and encodes a 170-kDa transmembrane tyrosine kinase receptor; EGFR is a member of the ErbB family of receptor tyrosinase kinases (TKIs), that includes ErbB2 (HER2 or Neu), ErbB3 (HER3) and ErbB4 (HER4) [25]. Binding of ligands like epidermal growth factor (EGF) or transforming growth factor-α (TGF-α) leads to homo- or hetero-dimerization with another member of the Her family resulting in the activation of different pathways [25]. EGFR is of fundamental importance in the regulation of epithelial differentiation and proliferation [26]. Recently, genetic studies using comparative genomic FISH have shown amplification of whole chromosome 7 and the 7p11.2 region, including the *EGFR* gene, in a large number of melanoma cases [27]. Chromosome 7 gain has been associated with thicker lesions, reduced survival and has been shown to occur more frequently in melanoma metastases compared to primary melanomas, as observed in our cohort. [28]. Nevertheless, the clinical implications of these findings are not clear as no prognostic significance of *EGFR* gene amplification was found in some studies and contrasting results were published in others, often carried out with different approaches [27, 29].

Similar difficulties arise in the comprehension of the clinical implications of *CCDN1* amplification in MCM patients. CCND1 is member of the highly conserved cyclin family. Cyclins act as regulators of the CDK kinases, by forming complexes with the regulatory subunits of CDK4 or CDK6, whose activity is required for cell cycle G1/S transition. These complexes have been shown to interact with tumor suppressor retinoblastoma protein (RB1), which in turn is a crucial regulator of successive phases of the cell cycle progression, including G1/S and G2/M transitions [30]. In summary, the activity of RB1 is mainly regulated by the status of the upstream CDKN2A/CCND1 pathway [31]. Anti-proliferative stresses—including DNA damage, therapeutic agents, and anti-mitogens—increase the expression of CDKN2A followed by the dissociation of CDK4/CDK6-CCND1 complexes. The CDK4/6-CCND1 inactivation leads to the hypo-phosphorylation of RB1, resulting in the transcriptional repression of E2F/DP regulatory genes that contributes to the progression of cell cycles. Data show that gene-silencing hyper-methylation in the promoter region of *CDKN2A* or overexpression of *CCND1* frequently occurs in several cancer types [32]. Our group previously evidenced that 13.6% of patients with multiple primary melanomas carry a *CCND1* gene amplification, with its higher incidence in subsequent lesions rather than in the primary ones [13]. Other studies reported a varying prevalence of *CCND1* amplification in MCMs [29, 33]. This heterogeneity in gene alteration distribution was somehow confirmed in the current study; *CCND1* amplification was observed in 25% of cell lines deriving from both primary and metastatic lesions but in 6% only of the *in vivo* melanoma human tissues.

Amplification of *cKIT* and *cMET* genes was exclusively found in cell lines obtained from metastasis, though they were found in both primary and metastatic human MCM tissues with no statistically significant difference between the two tissue types. The incidence of MCM cases with *cKIT* amplification ranges from 5% to 7% in previous reports [9, 13, 34]. Higher frequencies have been found in particular types of MCM, with the mucosal and acral melanomas carrying higher levels of copy number imbalances and mutations in the *cKIT* gene [34–35].

Our findings, when considered globally, outline the heterogeneous contribution of the different molecular impairments in terms of gene copy number variations to melanoma onset. Conversely, patterns of activating mutations in the main oncogenes involved in melanoma pathogenesis were confirmed to poorly vary during the natural history of MCM (i.e. moving from primary to metastatic lesions). Therefore, data here presented seem to suggest that acquisition of genomic amplifications affecting a panel of genes widely demonstrated to contribute to MCM pathogenesis may be strongly favored during the late phases of disease progression. In other words, one can speculate that while mutations in oncogenes and tumor suppressor genes may always act as driver alterations gene amplifications and/or gene copy number variations may prevalently act as passenger alteration in melanoma pathogenesis.

## MATERIALS AND METHODS

### Melanoma cell lines

Melanoma cell lines were kindly provided by Dr. Stefania D’Atri at the Institute *Dermopatico dell’Immacolata* in Rome. They were established as primary short term cell cultures, starting from primary and metastatic tumors samples of donor patients with documented diagnosis of melanoma, after obtaining their informed consent. Cell lines were maintained in RPMI medium supplemented with 10% fetal bovine serum, L-glutamine and penicilline/streptomycin. All chemicals were Reagent Grade and obtained from Sigma-Aldrich (Sigma-Aldrich, United States).

### Human melanoma samples

Patients with histologically-proven diagnosis of melanoma, regardless the disease stage at the time of diagnosis, were included into the study. A total of 274 formalin-fixed and paraffin embedded (FFPE) tumor tissues were collected from pathological archives of 232 consecutively-collected melanoma patients (for 42 of them, paired samples of primary melanomas and synchronous or asynchronous metastases were available).

### Mutation screening of candidate genes

For mutation analysis, genomic DNA was isolated from either melanoma cell lines or melanoma tissues, using standard methods. The full coding sequences and splice junctions of the *CDKN2A* (exons 1α, 2 and 3) and *NRAS* (exons 2-4) genes as well as the kinase domains of the *BRAF* (exons 11 and 15, where nearly all of the oncogenic mutations are located) gene were screened for mutations using an automated fluorescence-cycle sequencer (ABIPRISM 3130, Life Technologies-ThermoFisher Scientific, Waltham, MA, USA). Primer sets and protocols for polymerase chain reaction (PCR) assays were previously described [36].

All mutations detected in this study have previously been reported in the Human Gene Mutation Database (HGMD) at http://www.hgmd.cf.ac.uk/ac/index.php and in the Catalogue Of Somatic Mutations In Cancer (COSMIC) at http://www.sanger.ac.uk/genetics/CGP/cosmic/.

### Fluorescence *in situ* hybridization (FISH) analysis in interphase tumor cells

Fluorescence *in situ* hybridization (FISH) analysis was carried out in interphase tumor cells using the following BAC probes: RP11.215K24 (*MITF*; chromosome 3p14.1), CTD.2199A14 (*EGFR*; 7p11.2), RP11.300I6 (*Cyclin D1*/*CCND1*; 11q13.2), CTB.13N12 (*cMET*; 7q31.2), and RP11.586A2 (*cKIT*; 4q12). Specific probes for candidate genes and control centromeres were labeled with Spectrum-Orange and Spectrum-Green (Vysis, Downer’s Grove, IL, USA respectively), following the protocols previously described by our group [37, 38].

For FISH-based classification of each sample, 200 well-preserved and non-overlapping tumor cells were evaluated by at least two investigators, using an epi-fluorescence microscope (Olympus BX61) equipped with selective filters for the detection of Spectrum-Green, Spectrum-Orange, and DAPI.30. Three color images were captured using a digital imaging analysis system Cytovision.

Gene amplification was defined by: *a*) candidate gene to control centromere ratio > 2, according to the main criterion provided for assessing *EGFR* gene copy number in non small cell lung cancer (NSCLC) [39]; *b*) presence of at least a tetrasomic signal (> 2.0 gene copies per control centromere) in more than 15% of cells. The coexistence of both criteria indicated the occurrence of a high level of gene amplification. Specimens presenting with gene/centromere ratio < 2 or < 15% of cells displaying at least 4 copies of the gene signals were classified as disomic.

### Statistical analysis

Univariate analysis of the occurrence of gene alterations versus the number of lesions from metastatic sites and primary melanoma locations was performed by Pearson’s Chi-Square test, using the statistical package SPSS/7.5 for Windows.

## SUPPLEMENTARY MATERIALS TABLE





## Abbreviations

CDK4/6-CCND1: cyclin-dependent kinase 4/6-Cyclin D1
COSMIC: catalogue of somatic mutations in cancer
FFPE: formalin-fixed paraffin-embedded
FISH: fluorescence *in situ* hybridization
HDAC: histone deacetylase
HGMD: human gene mutation database
MCM: malignant cutaneous melanoma
MAPK: *mitogen-activated protein kinase*
MITF: microphthalmia-associated transcription factor
PCR: polymerase chain reaction
PI3K: phosphatidylinositol 3 kinase
TKIs: tyrosinase kinases
