# Supplementary material for: Genetic alterations in main candidate genes during melanoma progression
Source: Oncotarget. 2018 Jan 3;9(9):8531–41. doi: 10.18632/oncotarget.23989 (PMC5823576; doi:10.18632/oncotarget.23989)
Supplement: Supplementary file 1 [file oncotarget-09-8531-s001.pdf]

Genetic alterations in main candidate genes during melanoma progression

SUPPLEMENTARY MATERIALS

Supplementary Table 1: Percentages of gene alterations in melanoma cell lines. In bold, cases with gene amplification (according to the criteria reported in Materials and Methods). See Supplementary_ Table_1.
