# Supplementary material for: Genetic alterations in main candidate genes during melanoma progression
Source: Oncotarget. 2018 Jan 3;9(9):8531–41. doi: 10.18632/oncotarget.23989 (PMC5823576; doi:10.18632/oncotarget.23989)
Supplement: Supplementary file 2 [file oncotarget-09-8531-s002.docx]

**SUPPLEMENTARY TABLE 1.** Percentages of gene alterations in melanoma cell lines. In bold, cases with gene amplification (according to the criteria reported in Materials and Methods).

| **Cell line** | **Ratio** | **MITF (RP11.215K24; 3p14.1)** | | | | | **Ratio** | **CYCLIN D1 (RP11.300I6; 11q13.2)** | | | | |
| --- | --- | --- | --- | --- | --- | --- | --- | --- | --- | --- | --- | --- |
|  |  | disomy | trisomy | tetra-somy | penta-somy | > 6 copies |  | disomy | trisomy | tetra-somy | penta-somy | > 6 copies |
| GR-MEL | 1,20 | 60% | 40% |  |  |  | **2,20** | **10%** | **20%** | **20%** | **20%** | **30%** |
| LCP-MEL | 1,20 | 60% | 40% |  |  |  | 1,10 | 80% | 20% |  |  |  |
| LCM-MEL | 1,30 | 50% | 40% | 10% |  |  | 1,30 | 50% | 40% | 10% |  |  |
| PNP-MEL | 1,10 | 80% | 20% |  |  |  | 1,10 | 80% | 20% |  |  |  |
| PNM-MEL | 1,15 | 70% | 30% |  |  |  | 1,15 | 70% | 30% |  |  |  |
| WM-115-4 | 1,10 | 80% | 20% |  |  |  | **1,53** | **45%** | **20%** | **20%** | **15%** |  |
| WM-266-4 | **1,50** | **40%** | **25%** | **30%** | **5%** |  | 1,25 | 60% | 30% | 10% |  |  |
| PE-MEL-41 | **1,53** | **30%** | **35%** | **35%** |  |  | 1,23 | 60% | 35% | 5% |  |  |
| PE-MEL 43 | **1,50** | **35%** | **30%** | **35%** |  |  | 1,33 | 50% | 35% | 15% |  |  |
| PE-MEL 47 | **1,85** | **20%** | **30%** | **20%** | **20%** | **10%** | **1,85** | **20%** | **20%** | **30%** | **30%** |  |
| A375 | 1,15 | 70% | 30% |  |  |  | 1,08 | 85% | 15% |  |  |  |
| 397-MEL | **1,50** | **30%** | **40%** | **30%** |  |  | 1,10 | 80% | 20% |  |  |  |
| 13443-MEL | 1,15 | 70% | 30% |  |  |  | 1,13 | 75% | 25% |  |  |  |
| CN-MEL | 1,30 | 50% | 40% | 10% |  |  | 1,15 | 70% | 30% |  |  |  |
| MAR-MEL | ***3,00*** | ***0%*** | ***0%*** | ***0%*** | ***0%*** | ***100%*** | **1,80** | **30%** | **20%** | **20%** | **20%** | **10%** |
| MNG-MEL | 1,20 | 60% | 40% |  |  |  | 1,10 | 80% | 20% |  |  |  |
| CR-MEL | **1,40** | **40%** | **40%** | **20%** |  |  | ***2,00*** | ***10%*** | ***20%*** | ***40%*** | ***20%*** | ***10%*** |
| GL-MEL | 1,30 | 40% | 60% |  |  |  | 1,10 | 80% | 20% |  |  |  |
| PR-MEL | **1,55** | **30%** | **30%** | **40%** |  |  | 1,20 | 60% | 40% |  |  |  |
| SN-MEL | 1,13 | 75% | 25% |  |  |  | 1,20 | 60% | 40% |  |  |  |
| SK-MEL-28 | ***2,08*** | ***5%*** | ***30%*** | ***30%*** | ***15%*** | ***20%*** | **1,50** | **30%** | **40%** | **30%** |  |  |
| LB-24-MEL | ***2,44*** | ***2%*** | ***8%*** | ***30%*** | ***20%*** | ***40%*** | ***2,34*** | ***2%*** | ***8%*** | ***40%*** | ***20%*** | ***30%*** |
| M14 | 1,25 | 60% | 30% | 10% |  |  | 1,15 | 70% | 30% |  |  |  |
| SBCL2 | **1,40** | **45%** | **30%** | **25%** |  |  | 1,15 | 70% | 30% |  |  |  |
| ST-MEL | **1,40** | **40%** | **40%** | **20%** |  |  | 1,15 | 70% | 30% |  |  |  |
| UACC 62 | 1,10 | 80% | 20% |  |  |  | 1,28 | 60% | 25% | 15% |  |  |
| UACC 257 | ***3,00*** | ***0%*** | ***0%*** | ***0%*** | ***0%*** | ***100%*** | **1,70** | **20%** | **40%** | **20%** | **20%** |  |
| ME 33797 | **1,50** | **35%** | **40%** | **15%** | **10%** |  | 1,15 | 70% | 30% |  |  |  |
| COPA 159 | ***2,55*** | ***5%*** | ***10%*** | ***10%*** | ***20%*** | ***55%*** | 1,28 | 60% | 25% | 15% |  |  |
| MEL 3.0 | ***2,00*** | ***25%*** | ***15%*** | ***20%*** | ***15%*** | ***25%*** | 1,20 | 60% | 40% |  |  |  |
| INT 9009 | 1,15 | 70% | 30% |  |  |  | 1,28 | 60% | 25% | 15% |  |  |
| MALME 37 | 1,15 | 70% | 30% |  |  |  | 1,15 | 70% | 30% |  |  |  |
|  |  |  |  |  |  |  |  |  |  |  |  |  |
| **Cell line** | **Ratio** | **EGFR (CTD.2199A14; 7p11.2)** | | | | | **Ratio** | **cKIT (RP11.586A2; 4q12)** | | | | |
|  |  | disomy | trisomy | tetra-somy | penta-somy | > 6 copies |  | disomy | trisomy | tetra-somy | penta-somy | > 6 copies |
| GR-MEL | 1,27 | 60% | 27% | 13% |  |  | 1,15 | 70% | 30% |  |  |  |
| LCP-MEL | 1,28 | 60% | 25% | 15% |  |  | 1,05 | 90% | 10% |  |  |  |
| LCM-MEL | ***2,20*** | ***10%*** | ***20%*** | ***20%*** | ***20%*** | ***30%*** | 1,10 | 80% | 20% |  |  |  |
| PNP-MEL | 1,10 | 80% | 20% |  |  |  | 1,10 | 80% | 20% |  |  |  |
| PNM-MEL | **1,55** | **30%** | **40%** | **20%** | **10%** |  | 1,15 | 70% | 30% |  |  |  |
| WM-115-4 | ***2,00*** | ***20%*** | ***20%*** | ***20%*** | ***20%*** | ***20%*** | 1,15 | 70% | 30% |  |  |  |
| WM-266-4 | 1,20 | 60% | 40% |  |  |  | **1,60** | **40%** | **20%** | **20%** | **20%** |  |
| PE-MEL-41 | 1,20 | 60% | 40% |  |  |  | 1,13 | 75% | 25% |  |  |  |
| PE-MEL 43 | 1,30 | 40% | 60% |  |  |  | 1,10 | 80% | 20% |  |  |  |
| PE-MEL 47 | 1,31 | 50% | 38% | 12% |  |  | 1,13 | 75% | 25% |  |  |  |
| A375 | 1,15 | 70% | 30% |  |  |  | 1,10 | 80% | 20% |  |  |  |
| 397-MEL | 1,18 | 65% | 35% |  |  |  | 1,08 | 85% | 15% |  |  |  |
| 13443-MEL | ***2,35*** | ***10%*** | ***10%*** | ***20%*** | ***20%*** | ***40%*** | **1,60** | **40%** | **20%** | **20%** | **20%** |  |
| CN-MEL | 1,15 | 70% | 30% |  |  |  | 1,05 | 90% | 10% |  |  |  |
| MAR-MEL | ***2,55*** | ***0%*** | ***10%*** | ***20%*** | ***20%*** | ***50%*** | 1,10 | 80% | 20% |  |  |  |
| MNG-MEL | 1,15 | 70% | 30% |  |  |  | 1,05 | 90% | 10% |  |  |  |
| CR-MEL | 1,13 | 75% | 25% |  |  |  | 1,10 | 80% | 20% |  |  |  |
| GL-MEL | 1,25 | 60% | 30% | 10% |  |  | 1,08 | 85% | 15% |  |  |  |
| PR-MEL | 1,20 | 60% | 40% |  |  |  | 1,05 | 90% | 10% |  |  |  |
| SN-MEL | ***2,35*** | ***10%*** | ***10%*** | ***20%*** | ***20%*** | ***40%*** | 1,10 | 80% | 20% |  |  |  |
| SK-MEL-28 | **1,55** | **50%** | **20%** | **10%** | **10%** | **10%** | **1,53** | **40%** | **30%** | **15%** | **15%** |  |
| LB-24-MEL | **1,50** | **30%** | **40%** | **30%** |  |  | 1,10 | 80% | 20% |  |  |  |
| M14 | 1,20 | 60% | 40% |  |  |  | 1,13 | 75% | 25% |  |  |  |
| SBCL2 | ***2,05*** | ***20%*** | ***20%*** | ***20%*** | ***10%*** | ***30%*** | 1,05 | 90% | 10% |  |  |  |
| ST-MEL | 1,20 | 60% | 40% |  |  |  | 1,20 | 60% | 40% |  |  |  |
| UACC 62 | 1,15 | 70% | 30% |  |  |  | 1,15 | 70% | 30% |  |  |  |
| UACC 257 | ***2,20*** | ***10%*** | ***20%*** | ***20%*** | ***20%*** | ***30%*** | **1,35** | **48%** | **34%** | **18%** |  |  |
| ME 33797 | 1,10 | 80% | 20% |  |  |  | 1,10 | 80% | 20% |  |  |  |
| COPA 159 | 1,10 | 80% | 20% |  |  |  | 1,05 | 90% | 10% |  |  |  |
| MEL 3.0 | 1,30 | 50% | 40% | 10% |  |  | 1,13 | 75% | 25% |  |  |  |
| INT 9009 | **1,55** | **50%** | **20%** | **10%** | **10%** | **10%** | 1,08 | 85% | 15% |  |  |  |
| MALME 37 | **1,80** | **30%** | **20%** | **20%** | **20%** | **10%** | 1,20 | 60% | 40% |  |  |  |
|  |  |  |  |  |  |  |  |  |  |  |  |  |
| **Cell line** | **Ratio** | **cMET (CTB.13N12; 7q31.2)** | | | | |  |  |  |  |  |  |
|  |  | disomy | trisomy | tetra-somy | penta-somy | > 6 copies |  |  |  |  |  |  |
| GR-MEL | 1,13 | 75% | 25% |  |  |  |  |  |  |  |  |  |
| LCP-MEL | 1,13 | 75% | 25% |  |  |  |  |  |  |  |  |  |
| LCM-MEL | **1,55** | **30%** | **30%** | **40%** |  |  |  |  |  |  |  |  |
| PNP-MEL | 1,15 | 70% | 30% |  |  |  |  |  |  |  |  |  |
| PNM-MEL | ***2,35*** | ***0%*** | ***20%*** | ***20%*** | ***30%*** | ***30%*** |  |  |  |  |  |  |
| WM-115-4 | 1,10 | 80% | 20% |  |  |  |  |  |  |  |  |  |
| WM-266-4 | 1,15 | 70% | 30% |  |  |  |  |  |  |  |  |  |
| PE-MEL-41 | **1,50** | **30%** | **40%** | **30%** |  |  |  |  |  |  |  |  |
| PE-MEL 43 | 1,15 | 70% | 30% |  |  |  |  |  |  |  |  |  |
| PE-MEL 47 | 1,08 | 85% | 15% |  |  |  |  |  |  |  |  |  |
| A375 | 1,10 | 80% | 20% |  |  |  |  |  |  |  |  |  |
| 397-MEL | 1,15 | 70% | 30% |  |  |  |  |  |  |  |  |  |
| 13443-MEL | **1,80** | **30%** | **20%** | **20%** | **20%** | **10%** |  |  |  |  |  |  |
| CN-MEL | 1,15 | 70% | 30% |  |  |  |  |  |  |  |  |  |
| MAR-MEL | **1,85** | **20%** | **20%** | **30%** | **30%** |  |  |  |  |  |  |  |
| MNG-MEL | 1,13 | 75% | 25% |  |  |  |  |  |  |  |  |  |
| CR-MEL | 1,15 | 70% | 30% |  |  |  |  |  |  |  |  |  |
| GL-MEL | 1,15 | 70% | 30% |  |  |  |  |  |  |  |  |  |
| PR-MEL | 1,05 | 90% | 10% |  |  |  |  |  |  |  |  |  |
| SN-MEL | 1,15 | 70% | 30% |  |  |  |  |  |  |  |  |  |
| SK-MEL-28 | **1,53** | **45%** | **20%** | **20%** | **15%** |  |  |  |  |  |  |  |
| LB-24-MEL | **1,50** | **35%** | **30%** | **35%** |  |  |  |  |  |  |  |  |
| M14 | 1,10 | 80% | 20% |  |  |  |  |  |  |  |  |  |
| SBCL2 | 1,10 | 80% | 20% |  |  |  |  |  |  |  |  |  |
| ST-MEL | 1,10 | 80% | 20% |  |  |  |  |  |  |  |  |  |
| UACC 62 | 1,10 | 80% | 20% |  |  |  |  |  |  |  |  |  |
| UACC 257 | 1,08 | 85% | 15% |  |  |  |  |  |  |  |  |  |
| ME 33797 | 1,15 | 70% | 30% |  |  |  |  |  |  |  |  |  |
| COPA 159 | 1,10 | 80% | 20% |  |  |  |  |  |  |  |  |  |
| MEL 3.0 | 1,10 | 80% | 20% |  |  |  |  |  |  |  |  |  |
| INT 9009 | 1,10 | 80% | 20% |  |  |  |  |  |  |  |  |  |
| MALME 37 | 1,20 | 60% | 40% |  |  |  |  |  |  |  |  |  |
